# Impact of isoflavone genistein on psoriasis in in vivo and in vitro investigations

**DOI:** 10.1038/s41598-021-97793-4

**Published:** 2021-09-14

**Authors:** Katarzyna Bocheńska, Marta Moskot, Elwira Smolińska-Fijołek, Joanna Jakóbkiewicz-Banecka, Aneta Szczerkowska-Dobosz, Bartosz Słomiński, Magdalena Gabig-Cimińska

**Affiliations:** 1 Department of Medical Biology and Genetics, University of Gdańsk, Wita Stwosza 59, 80-308 Gdańsk, Poland; 2 Institute of Biochemistry and Biophysics, Laboratory of Molecular Biology, Polish Academy of Sciences, Kładki 24, 80-822 Gdańsk, Poland; 3 Department of Dermatology, Venereology and Allergology, Medical University of Gdańsk, Mariana Smoluchowskiego 17, 80-214 Gdańsk, Poland; 4 Department of Immunology, Faculty of Medicine, Medical University of Gdańsk, Dębinki 1, 80-211 Gdańsk, Poland

**Keywords:** Cell biology, Molecular biology, Diseases, Skin diseases

## Abstract

Genistein is applied worldwide as an alternative medicament for psoriasis (Ps) because of its anti-inflammatory activity and perceived beneficial impact on the skin. Hereby, we report our in vivo and in vitro investigations to supplement scientific research in this area. The reduction of clinical and biochemical scores in mild to moderate Ps patients taking genistein, its safety, good tolerability with no serious adverse events or discontinuations of treatment, no dose-limiting toxicities, negligible changes in pharmacodynamic parameters and remarkable serum interleukin level alterations were documented in this study. A certain regression of the Ps phenotype was visible, based on photo-documented Ps lesion evaluation. Through in vitro experiments, we found that genistein reduced IL-17A and TNF-α induced MAPK, NF-κB, and PI3K activation in normal human epidermal keratinocytes. Moreover, at the mRNA level of genes associated with the early inflammatory response characteristic for Ps (*CAMP*, *CCL20*, *DEFB4A*, *PIK3CA*, *S100A7*, and *S100A9*) and key cellular signalling (*MTORC1* and *TFEB*), we showed that this isoflavone attenuated the increased response of IL-17A- and TNF-α-related pathways. This allows us to conclude that genistein is a good candidate for Ps treatment, being attractive for co-pharmacotherapy with other drugs.

## Introduction

Psoriasis (Ps) is a disease of complex aetiology in which genetic (more than 60 loci significantly associated with Ps susceptibility identified by genome-wide association study (GWAS) analyzes—the PSORS1 locus on chromosome 6p21 within the MHC region shows the strongest linkage with Ps), as well as genomic (susceptibility determined by many genes, even by entirety of the person’s genes, including interactions of those genes with each other and with the person's environment; around 9 000 differentially regulated transcripts between psoriatic lesional (PP), psoriatic non-lesional (PN), and normal control skin (NN) identified by RNA-Seq analyzes) and immunological factors (e.g., autoantigens, proinflammatory cytokines, activated immune cells) play a major role. Conversely, environmental causes, such as stress (e.g. through dysregulation of local neuroendocrine-immune activities and corticotropin-releasing hormone, CRF, signaling), infections (incl. *Staphylococcus aureus* or *Streptococcus pyogenes*), mechanical injuries, or some medicines (such as lithium, beta-blockers, antimalarial drugs, and imiquimod), are most likely responsible for the onset and recurrence of the disease. Up to 30% of patients with chronic plaque Ps are also diagnosed with psoriatic arthritis^1–3^. The excessive number of keratinocyte divisions in the basal layer of the epidermis and their accelerated abnormal maturation cycle is responsible for the manifestation of characteristic disease symptoms. Formation of the proper skin barrier in Ps is impaired, among others, by suppression of glucocorticoid biosynthesis by pro-inflammatory cytokines^4^. Pathogenesis of Ps includes interactions between different types of immune cells and cytokines, resulting in overstimulation and dysregulation of the immune system. Cytokines, such as interferon gamma (IFN-γ), tumor necrosis factor-alpha (TNF-α), and interleukins 12 (IL-12), 17 (IL-17), and 23 (IL-23), are mainly involved in the pathophysiology of Ps. IL-12 influences the development of CD4 + T helper (Th) 1 cells and CD8 + T effector cytotoxic (Tc) cells. In turn, IL-23 is responsible for the differentiation of CD4 + precursor cells into Th17 cells. Moreover, IL-23 together with TNF-α is responsible for the proliferation and survival of Th17 lymphocytes. It is widely believed that IL-23 is necessary for the full inflammatory function of Th17 lymphocytes related to the secretion of cytokines, including IL-17. Increasing level of IL-17 induces inflammation and the development of Ps skin lesions due to the hypertrophy of the epidermis and the excessive proliferation of keratinocytes^5,6^. TNF-α and IL-17 receptors are localised on keratinocyte surfaces^7,8^. Their stimulation by TNF-α and IL-17 results in greater changes in gene expression encoding inflammatory products at synergistic levels than either alone. Therefore, most important immune products are under control by TNF-α/IL-17 synergism^9^.

In mild Ps, which affects about 85% of cases, topical treatment with tar, cygnoline, corticosteroids, vitamin D derivatives or retinoids is used. In turn, due to the significant efficiency and relatively high safety profile of biological drugs, these compounds are increasingly used in the treatment of severe Ps. Considering their structure, monoclonal antibodies, fusion proteins and recombinant human proteins (including TNF-α inhibitors, IL-12/IL-23 inhibitors, drugs that inhibit the proliferation of T lymphocytes or drugs that reduce the activation and migration of T lymphocytes) are distinguished. However, small molecule therapy is not available to every patient—the most common contraindications include viral hepatitis, chronic inflammation, pregnancy and breastfeeding, connective tissue diseases, active neoplastic diseases, active bacterial, viral and fungal infections^10,11^. Unfortunately, due to concomitant diseases and differences among individuals, medications used in Ps are not always safe and often ineffective; therefore, scientists are looking for alternative, harmless, and natural therapeutic sources. The lack of effective Ps therapies without restrictive side effects is still a problem for more than 125 million people worldwide, according to the World Psoriasis Day consortium data^12^. Ps confers significant physical and psychological distress and impairment, usually resulting in a detrimental impact on the patient’s quality-of-life. Patients often express feelings of shame and guilt and are stigmatised by the disease. About 80% of patients reported that Ps had a negative impact on their lives, while 40% of individuals with severe disease were frustrated with the ineffectiveness, cost, and/or inconvenience of their current therapies. A total of 32% viewed their therapy as unsatisfactory^13^.

Polyphenols, and among them, isoflavones, belonging to secondary metabolites of plants, are well-known for their antioxidant and antiradical properties^14^. Due to the wide range of pharmacological effects of isoflavones, attempts to use these compounds as adjunct therapy in many diseases have also been made. Research indicates the ability of plant-derived substances to modulate the function of the human immune system by affecting the proliferation of immune cells and the production of cytokines or other factors involved in defence reactions. These compounds are considered regulators of inflammatory responses in the skin and can consequently be useful as a part of the treatment of atopic dermatitis, vitiligo, and Ps^15–17^. Increasing knowledge about the mechanism of action of one of the isoflavones, genistein, has contributed to its application in inflammatory skin disorders^18^. Genistein (5,7-dihydroxy-3-(4-hydroxyphenyl) chromen-4-one) is the major metabolite of soy with a 15-carbon skeleton and a chemical structure resembling estradiol, leading to its binding to human estrogen receptor α (ERα) and β (ERβ)^19,20^. Notably, the intake of genistein, even at relatively high doses, is safe and should not cause any side effects. Only a few studies have indicated its potential minimal toxicity (clinically manifested, inter alia, nausea, pedal edema or breast tenderness) at 16 mg/kg body weight doses^21^. The topical application of genistein to the skin has shown that it is moderately absorbed (the most in pH 6 buffer) and has almost no adverse effects like erythema or stratum corneum disruption^22^. In vivo studies using an animal model of rheumatoid arthritis showed that genistein inhibited IFN-γ secretion and simultaneously enhanced interleukin 4 (IL-4) production by peripheral blood mononuclear cells (PBMCs), which contributed to maintaining a balance between T helper type 1 (Th1) and 2 (Th2) responses^23,24^. The combination of anti-inflammatory action and the inhibition of cell proliferation by genistein can be very promising in the treatment of Ps. These effects may be complementary to systemic treatment, but genistein can also be used as a substantive therapy for milder forms of this disease.

## Results

### In vivo studies

#### Safety outcomes

**Figure 1:**
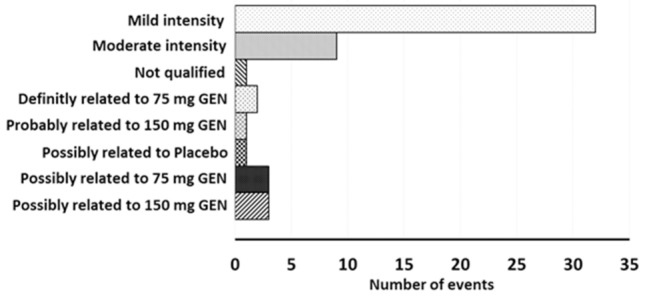
The most frequent adverse events (AEs) reported in the study due to all causes. AEs are schematised according to their causality and severity. The most common AEs reported were of mild intensity (*n* = 32). Only three severe AEs were definitely related to the study treatment in the 75 mg genistein group.

Genistein was generally well tolerated by 24 of 40 randomised patients and no serious adverse events (AEs) or discontinuation of treatment occurred. The total number of adverse events reported for the duration of the study was 42, of which the majority (78%; *n* = 32) were mild in intensity (e.g., cold, herpes simplex, oral candidiasis, right heel pain), and 22% (*n* = 9) were moderate in intensity (e.g., headache, sinusitis, rash, pharyngitis). Only one AE was not qualified. Two AEs (4.8%) were definitely related to study treatment (all 2 in the genistein 75 mg/dose group), one AE (2.4%) was probably related to study treatment (in the genistein 150 mg/dose group), and seven AEs (16.7%) were possibly related to study treatment (three AEs in the genistein 75 mg/dose group, three AEs in the genistein 150 mg/dose group, and one AE in the placebo group) (Fig. 1).

#### Clinical response to genistein treatment

Among the 49 patients who were screened, 40 with mild to moderate chronic plaque psoriasis (Ps) were enrolled and treated with blinded study drug, and the remaining nine people did not complete the test. Ten patients (10 completed) were randomised to placebo, 15 (12 completed) to the 75 mg/dose group, and 15 (12 completed) to the 150 mg/dose set. The 34 enrolled patients received all planned doses of the study drug or placebo and completed the study after 56 days. Although there was some variability, baseline demographics and clinical disease characteristics were generally similar across treatment groups. The age of patients in this study ranged from 30 to 62 years; 41% were female and 59% were male, giving a male to female ratio of 1.43:1, and all of them were Caucasian race. The baseline disease activity was typically mild to moderate in all three tested sets with the groups receiving active treatment trending toward greater activity in Psoriasis Area and Severity Index (PASI), Body Surface Area (BSA), and Physician’s Global Assessment (PGA) (Table 1). Except for the result, which was close to statistical significance (*p* = 0.0506) for the PGA score in the 75 and 150 mg/dose genistein groups (GEN 75 and GEN 150, respectively) and placebo on day 56, we did not observe any other significant changes.

**Table 1:** Changes in Psoriasis Area and Severity Index (PASI), Body Surface Area (BSA), and Physician’s Global Assessment (PGA) score from visit 1 (day 0) to visit 3 (day 56) according to treatment groups of 75 mg (GEN 75) or 150 mg (GEN 150) genistein administration, in a total of 34 from 40 randomised patients.

	PASI				BSA				PGA			
	GEN 75 *n* = 12	GEN 150 *n* = 12	Placebo *n* = 10	*p-*value	GEN 75 *n* = 12	GEN 150 *n* = 12	Placebo *n* = 10	*p-*value	GEN 75 *n* = 12	GEN 150 *n* = 12	Placebo *n* = 10	*p-*value
	Day 0			0.4353*	Day 0			0.5603**	Day 0			0.3406*
Mean (SD)	6.2 (2.7)	6.8 (2.3)	5.7 (2.6)		4.1 (2.4)	4.6 (2.1)	3.6 (2.3)		–	–	–	
95% CI	4.7; 7.7	5.5; 8.1	3.9; 7.6		2.7; 5.4	3.4; 5.8	2.0; 5.2		–	–	–	
Range (min–max)	1.4–11.7	4.2–11.6	2.4–10.7		1.2–9.8	1.4–7.5	0.7–8.0		2.0–3.0	2.0–3.0	2.0–2.0	
Median	5.9	5.8	4.9		3.2	4.3	3.0		2.0	2.0	2.0	
	Day 28			0.4104**	Day 28			0.3776**	Day 28			0.2231*
Mean (SD)	5.4 (2.4)	6.8 (3.7)	5.3 (3.1)		4.0 (2.4)	5.1 (2.7)	3.9 (2.3)		–	–	–	
95% CI	4.0; 6.8	4.5; 9.1	3.1; 7.5		2.6; 5.3	3.4; 6.8	2.3; 5.5		–	–	–	
Range (min–max)	1.8–11.5	2.3–14.9	1.7–10.7		1.2–9.8	1.6–10.5	1.3–8.0		2.0–3.0	1.0–3.0	1.0–2.0	
Median	5.8	5.9	4.6		3.2	5.6	3.5		2.0	2.0	2.0	
	Day 56			0.1376***	Day 56			0.3586**	Day 56			0.0506*
Mean (SD)	5.3 (2.1)	7.3 (4.3)	4.3 (2.1)		4.0 (2.4)	5.3 (2.8)	3.8 (2.6)		–	–	–	
95% CI	4.0; 6.5	4.5; 10.0	2.8; 5.8		2.6; 5.5	3.5; 7.1	2.0; 5.6		–	–	–	
Range (min–max)	2.0–8.8	2.3–16.8	1.8–8.4		1.2–9.0	1.1–10.5	1.0–8.5		2.0–3.0	1.0–3.0	1.0–2.0	
Median	5.3	5.6	3.7		2.9	5.0	3.4		2.0	2.0	2.0	
*p-*value	0.2404****	0.3067****	0.1390****		0.8232*****	0.7135*****	0.9094*****		0.5488****	0.7788****	0.1738****	

*P*-values close to statistical significance are shown in bold.

*Kruskal–Wallis; **Fischer test (ANOVA); ***Fischer Welch test (ANOVA); ****ANOVA Friedman; *****ANOVA.

**Figure 2:**
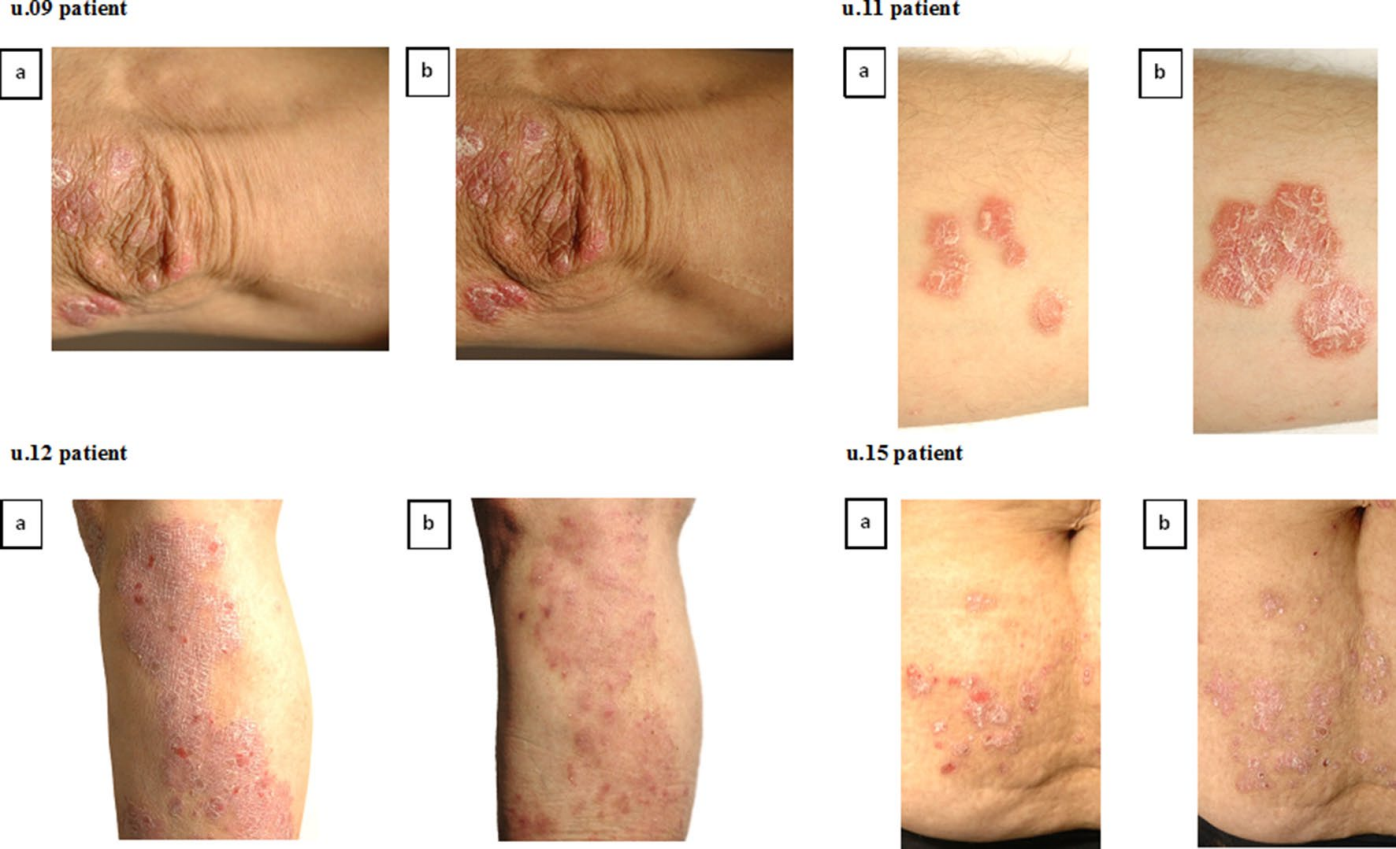
Individual genistein score over time. Specific genistein responses in four patients, named u.09, 11, 12 and 15, by baseline (**a**) and after 56 days of genistein or placebo treatment (**b**).

In addition, the impact of isoflavone genistein on Ps patients was minutely studied for 4 of the 34 enrolled (40 randomised) subjects with chronic plaque Ps, named u.09, 11, 12, and 15. These patients were included in both biochemical and genetic analyses, as described below. The average age of these subjects was 46 years (range 34–60), while 50% of the subjects were males. Subjects' body mass index (BMI) range was 26.5–33.5 kg/m^2^, height range was 159–178 cm, and weight range was 67–98 kg. All four subjects completed the study and were included in the outcome analyses. The baseline characteristics of these patients are summarised in Table 2. We detected more than a two-fold reduction in PASI, a slight decrease in BSA, and no changes in PGA in patients u.09 and 12, while there were no alterations for the other genistein-treated subject u.15 in these scores of the three measured clinical parameters, as was the case for the placebo patient (u.11). Similarly, when evaluating psoriatic lesions, documented in the form of photographic pictures from a particular part of the body of all tested patients, one can infer a certain regression of the psoriatic phenotype in the case of subject u.12 (Fig. 2).

**Table 2:** Characteristics of psoriasis patients u.09, 11, 12, and 15 in the present study.

Patient	Age	Sex	Race	Weight (kg)	BMI	PASI		BSA		PGA		Dose of genistein (mg)
						Day 0	Day 56	Day 0	Day 56	Day 0	Day 56	
u.09	51	Male	Caucasian	98	33.5	3.6	8.8	1.5	2.5	2	3	75
u.11	34	Male	Caucasian	94	29.7	7.0	8.4	3.0	8.5	2	2	0
u.12	60	Female	Caucasian	67	26.5	11.7	5.2	9.75	9.0	2	2	75
u.15	39	Female	Caucasian	80	29.4	9.20	16.8	7.5	10.5	2	3	150

#### Cytokine profile during genistein monotherapy

Pharmacodynamics results in genistein groups vs. placebo, including changes in the of cytokines, i.e., interleukin (IL)-12, IL-17, IL-23, and tumor necrosis factor-alpha (TNF-α), were as denoted in Table 3, in total for 34 of 40 randomised patients. Median values in all treatment at visits 1 and 3 (days 0 and 56, respectively) were as follows: for IL-12 < 3.1 pg/ml, IL-17 < 31.2 pg/ml, and for IL-23 < 15.6 pg/ml, except for the genistein 75 mg/dose group at visit 3 (15.9 pg/ml). TNF-α level medians were 5.3 pg/ml and 5.5 pg/ml at visits 1 and 3, respectively, in the genistein 75 mg/dose group, 5.7 pg/ml, 6.2 pg/ml at visits 1 and 3, respectively, in the 150 mg/dose group and were 5.0 pg/ml and 6.0 pg/ml at visits 1 and 3, respectively, in the placebo group. The obtained pharmacodynamics results were not statistically significant between treatment groups on days 0 and 56 and within treatment groups on days 0 and 56, except for the significant change (*p* = 0.0277) in IL-23 level in the placebo group. An increase in the highest level from 20.1 pg/ml on day 0 to 27.1 pg/ml on day 56 was detected.

**Table 3:** Pharmacodynamic results of the levels of serum cytokines and comparisons before (day 0) and after (day 56) 75 mg (GEN 75) or 150 mg (GEN 150) genistein administration and genistein-untreated placebo, in total, for 34 of the 40 randomised patients.

	Interleukin 12 (pg/ml)				Interleukin 17 (pg/ml)				Interleukin 23 (pg/ml)				Tumor necrosis factor-α (pg/ml)			
	GEN 75 *n* = 12	GEN 150 *n* = 12	Placebo *n* = 10	*p-*value	GEN 75 *n* = 12	GEN 150 *n* = 12	Placebo *n* = 10	*p-*value	GEN 75 *n* = 12	GEN 150 *n* = 12	Placebo *n* = 10	*p-*value	GEN 75 *n* = 12	GEN 150 *n* = 12	Placebo *n* = 10	*p-*value
	Day 0			1.00*	Day 0			0.1752*	Day 0			0.9122**	Day 0			0.8379**
Median	< 3.1	< 3.1	< 3.1		< 31.3	< 31.3	< 31.3		< 15.6	< 15.6	< 15.6		5.3	5.7	5.0	
Q1; Q3	< 3.1; < 3.1	< 3.1; < 3.1	< 3.1; < 3.1		< 31.3; < 31.3	< 31.3; < 31.3	< 31.3; < 31.3		< 15.6; < 15.6	< 15.6; < 15.6	< 15.6; < 15.6		4.9; 7.5	4.3–8.6	4.3–16.0	
Range (min–max)	< 3.1- < 3.1	< 3.1- < 3.1	< 3.1- < 3.1		< 31.3; < 31.3	< 31.3; < 31.3	< 31.3; < 31.3		< 15.6–36.1	< 15.6–22.0	< 15.6–20.0		< 4.0–8.8	4.3–8.6	4.3–16.0	
	Day 56			0.2865*	Day 56			1.00*	Day 56			0.8895*	Day 56			0.6401*
Median	< 3.1	< 3.1	< 3.1		< 31.3	< 31.3	< 31.3		15.9	< 15.6	< 15.6		5.5	6.2	6.0	
Q1; Q3	< 3.1; < 3.1	< 3.1; < 3.1	< 3.1; < 3.1		< 31.3; < 31.3	< 31.3; < 31.3	< 31.3; < 31.3		< 15.6; < 15.6	< 15.6; < 15.6	< 15.6; < 15.6		4.9; 7.4	5.6; 7.0	4.6; 6.2	
Range (min–max)	< 3.1; < 3.1	< 3.1; < 3.1	< 3.1; < 3.1		< 31.3; < 31.3	< 31.3; < 31.3	< 31.3; < 31.3		< 15.6–29.5	< 15.6–24.6	< 15.6–27.1		< 4.0–8.1	4.5–8.4	< 4.0–13.0	
*p-*value	n.d	n.d	n.d		0.1797**	n.d	n.d		0.1614*	0.1159**	0.0277**		0.3670*	0.1424**	0.0528**	

*P*-values of statistical significance are shown in bold.

*Kruskal–Wallis test; **Wilcoxon test.

n.d. shortcut stands for no data available.

Moreover, changes in interleukin levels in the serum of four patients (u.09, 11, 12, and 15) are shown in Table 4. Levels of IL-12 and IL-17 in all subjects at days 0 and 56 were as follows: < 3.1 pg/ml for IL-12 and < 31.2 pg/ml for IL-17. In patient u.09, we observed a decrease in the levels of IL-23 and TNF-α in the serum on treatment day 56 with 75 mg genistein. In patients u.11, u.12, and u.15, an increase in IL-23 was detected. The level of TNF-α was reduced at visit 3 compared to visit 1 for patient u.11, while for subjects u.12 and u.15, we observed subtle growth in the level of this serum cytokine.

**Table 4:** Changes in the levels of serum cytokines and comparisons before (day 0) and after (day 56) genistein administration in patients u.09, 12 and 15, and in genistein-untreated placebo u.11.

Visit	u.09	u.11	u.12	u.15
	**Interleukin 12 (pg/ml)**			
Day 0	< 3.1	< 3.1	< 3.1	< 3.1
Day 56	< 3.1	< 3.1	< 3.1	< 3.1
	**Interleukin 17 (pg/ml)**			
Day 0	< 31.1	< 31.1	< 31.1	< 31.1
Day 56	< 31.1	< 31.1	< 31.1	< 31.1
	**Interleukin 23 (pg/ml)**			
Day 0	36.1	< 15.6	22.0	< 15.6
Day 56	< 15.6	27.1	29.5	22.0
	**Tumor necrosis factor-α (pg/ml)**			
Day 0	4.9	7.4	7.5	4.7
Day 56	< 4.0	6.1	7.7	5.5

### In vitro experiments

#### Genistein affects the MAPK pathway phosphorylation status

**Figure 3:**
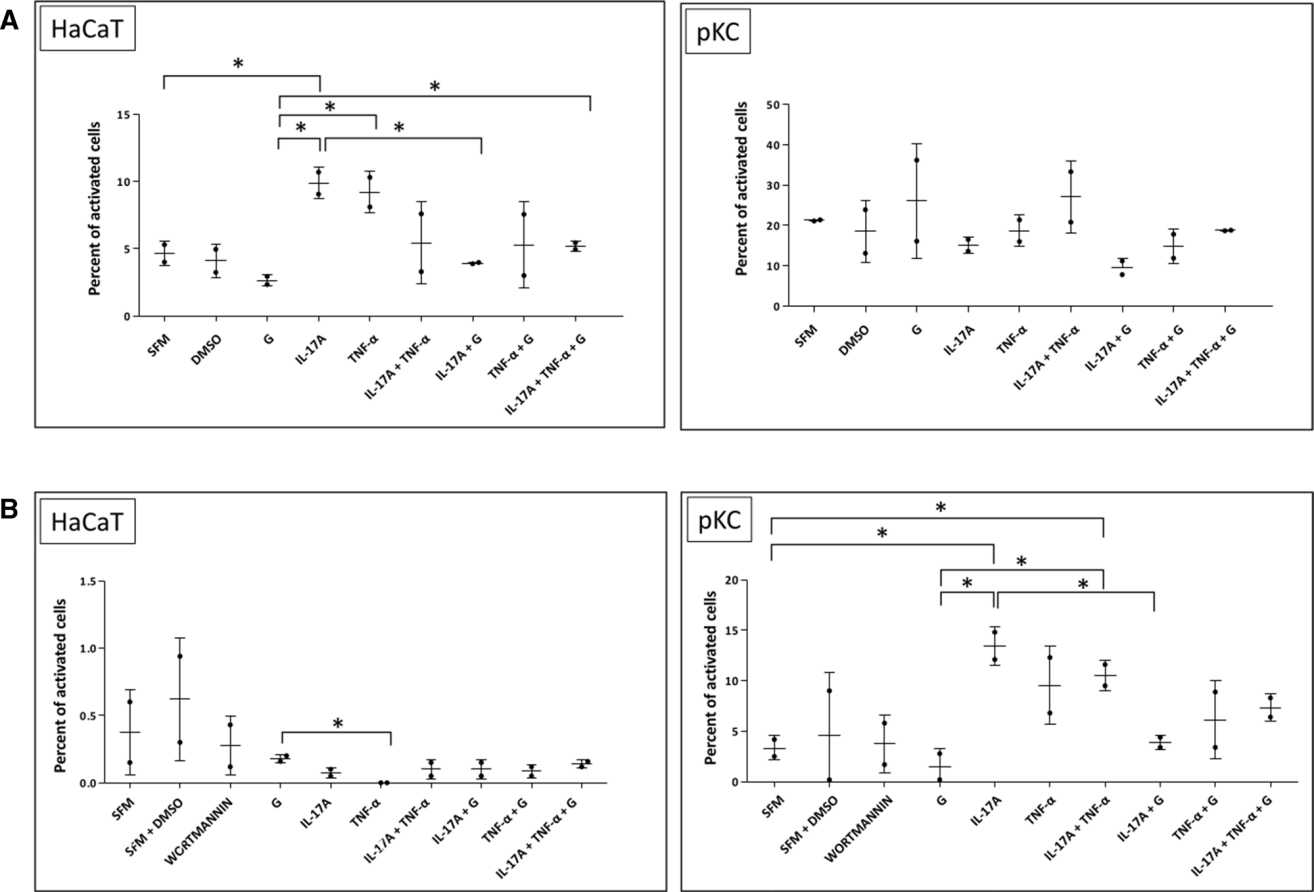
Effects of IL-17A, TNF-α, or an IL-17A/TNF-α mix and IL-17A, TNF-α, or an IL-17A/TNF-α mix with 100 μM genistein for 3 h on the modulation of MAPK ERK1/2 (**A**) and PI3K (**B**) signalling in HaCaTs and pKCs. Control cells were maintained in SFM with or without DMSO. Wortmannin was used as an additional control (PI3K inhibitor). Each dot represents the mean and error bars the standard deviation of two independent experiments (*n* = 2). For each independent experiment, the mean was calculated from duplicate measurements and used in subsequent calculations. Statistical differences were observed following application of a parametric Student's t-test, with continuity correction (*p* ≤ 0.05). The asterisks designate a statistically significant difference between the compared groups. *SFM* serum-free medium, *DMSO* dimethyl sulfoxide, *G* genistein, *IL-17A* interleukin-17A, *TNF-α* tumor necrosis factor-alpha.

To assess the activation of mitogen-activated protein kinase (MAPK) following exposure to IL-17A, TNF-α, and an IL-17A/TNF-α mix, we examined the phosphorylation level of extracellular signal-regulated kinase 1/2 (ERK1/2), as the relevant downstream molecule via Guava® Muse® Cell Analyzer analysis (Fig. 3A). MAPK signalling is critically involved in IL-17A and TNF-α-activated pathways and regulates various downstream effects in skin cells. In this study, therefore, we investigated possible modulation of this crucial signalling pathway by the isoflavone genistein. For both human adult low calcium temperature cells (HaCaTs) and primary keratinocytes (pKCs), ERK1/2 analyses were performed after the cells were pretreated with isoflavone for 1 h and stimulated with IL-17A, TNF-α, or IL-17A/TNF-α mix for the next 24 h. In two independent experiments, the statistical analysis performed by using a Student's t-test revealed significant alterations in MAPK activity; therefore, ERK1/2 phosphorylation, in IL-17A stimulated HaCaTs, followed by a decrease after exposure of these cells to genistein. No statistically important differences in MAPK activity in pKCs were observed, although the stimulation by applied cytokines was biased and prevented by pretreatment with isoflavone. An average of 10,000 cells were analysed for each condition. The percentage of inactivated cells, activated cells (via phosphorylation of MAPK relevant downstream molecule ERK1/2), and non-expressing cells was determined for each experimental condition.

#### Significant inhibition of IL-17A-induced PI3K activation

In this part of the work, Guava® Muse® Cell Analyzer analysis was done to study the activity of phosphoinositide 3-kinase (PI3K) in HaCaT and pKC pretreated with genistein for 1 h and stimulated with IL-17A, TNF-α, and IL-17A/TNF-α or incubated with 50 nM wortmannin, as an additional control (a PI3K inhibitor). The percentage of inactivated cells, activated cells (via PI3K phosphorylation), and non-expressing cells was determined for each experimental condition (Fig. 3B). The statistically important change in activated HaCaTs assessed by PI3K signalling was observed in cells treated with genistein compared to TNF-α. Additionally, a significant effect was noted for pKCs stimulated by IL-17A and the IL-17A/TNF-α mix with regard to vehicles (cells maintained only in serum-free medium—SFM). Notably, the statistical tests confirmed a significant IL-17 dependent increase on PI3K activity in pKCs (*p* < 0.0001), followed by its substantial reduction after the addition of genistein into the sample, indicating potential anti-inflammatory properties of the tested isoflavone.

#### Genistein modulates IL-17A, TNF-α, and IL-17A/TNF-α mix-induced NF-κB pathway activation

**Figure 4:**
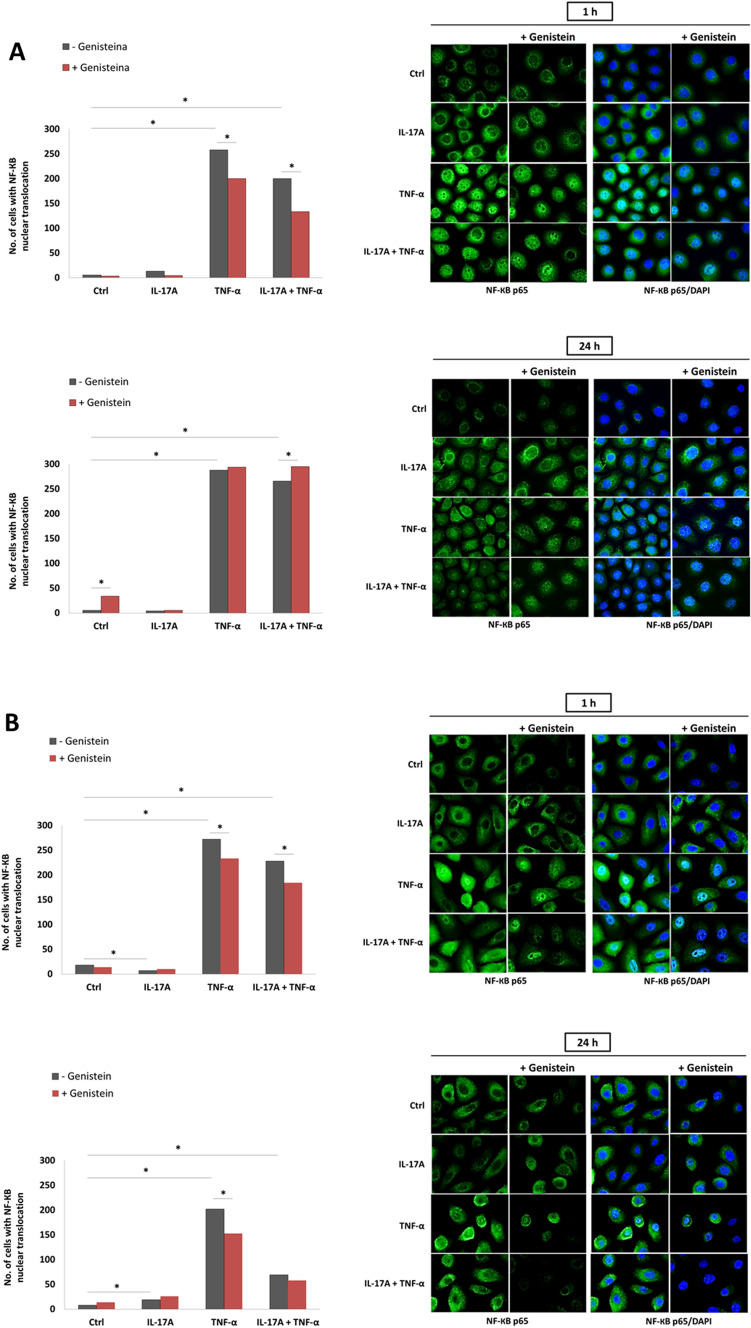
Intracellular localisation of the p65 subunit of NF-κB visualised with fluorescence microscopy in HaCaT (**A**) and pKC (**B**). Cells were pretreated with 100 μM genistein for 1 h, and then incubated with an IL-17A (100 ng/ml), TNF-α (10 ng/ml), or a mix of both for 1 or 24 h. The control cells were treated with 0.05% DMSO or with 100 μM genistein. Nuclei were counterstained with DAPI. Graphical representation depicted as the number of cells with NF-κB nuclear translocation; p65 labelling was shown in 300 cells from randomly selected, non-overlapping microscopic fields. The asterisks designate a statistically significant difference. *IL-17A* interleukin-17A, *TNF-α* tumor necrosis factor-alpha, *G* genistein, *NF-κB p65* nuclear factor kappa-light-chain-enhancer of activated B cells, p65 subunit.

Study of the nuclear translocation of the nuclear factor kappa-light-chain-enhancer of activated B cells, p65 subunit (NF-κB p65 subunit) in keratinocytes, HaCaTs, and pKCs, activated with IL-17A, TNF-α, or an IL-17A/TNF-α mix, using immunofluorescence microscopy with or without genistein treatment, revealed remarkable modulation of this process by the tested isoflavone (Fig. 4). In the case of HaCaTs, 85 and 65% of the cells displayed NF-κB p65 nuclear localisation after 1 h of TNF-α or IL-17A/TNF-α mix administration, respectively. These levels were decreased to 63 and 45% for samples pretreated with the isoflavone. A 24 h incubation with cytokines resulted in 95 and 88% translocation of NF-κB p65 into the nucleus in cells cultivated with TNF-α or an IL-17A/TNF-α mix, respectively. One hour pretreatment with genistein increased the level in a statistically significant manner (i.e., to 97%, only for the IL-17A/TNF-α mix). We did not find any statistically significant changes in relocation of NF-κB p65 from the cytosol to the nucleus when keratinocytes were exposed either for 1 or 24 h to IL-17A, as well as to IL-17A and genistein combination (Fig. 4A). For pKCs treated with cytokines TNF-α or the IL-17A/TNF-α mix for 1 h we observed nuclear translocation of NF-κB p65 for 90 and 75% of cells, respectively. One hour pretreatment with genistein reduced these levels to 77 and 62%, respectively. When incubated with IL-17A for 1 h, we noticed statistically important NF-κB p65 suppression in cytoplasm-to-nucleus shuttling. After 24 h of cytokine activation, 6, 67, and 22% of cells revealed NF-κB p65 translocation into the nucleus for IL-17A, TNF-α, and IL-17A/TNF-α mix, respectively, followed by a decrease to 50% for pKCs incubated with TNF-α and pretreated with genistein. Any statistically important change in NF-κB p65 transfer into the nucleus was observed after the addition of IL-17A and genistein combination compared to IL-17A treated pKCs, irrespective of the treatment time (Fig. 4B).

Moreover, our analyses also revealed a decrease in the cytoplasmic level of NF-κB p65 in pKCs after 1 h genistein application for all conditions and treatment times. However, in the case of HaCaTs, there was no apparent difference in the NF-κB p65 cytoplasmic level.

#### Genistein attenuates IL-17A, TNF-α, and IL-17A/TNF-α mix-induced mRNA expression of the early inflammatory response and key cellular signalling genes tested

**Figure 5:**
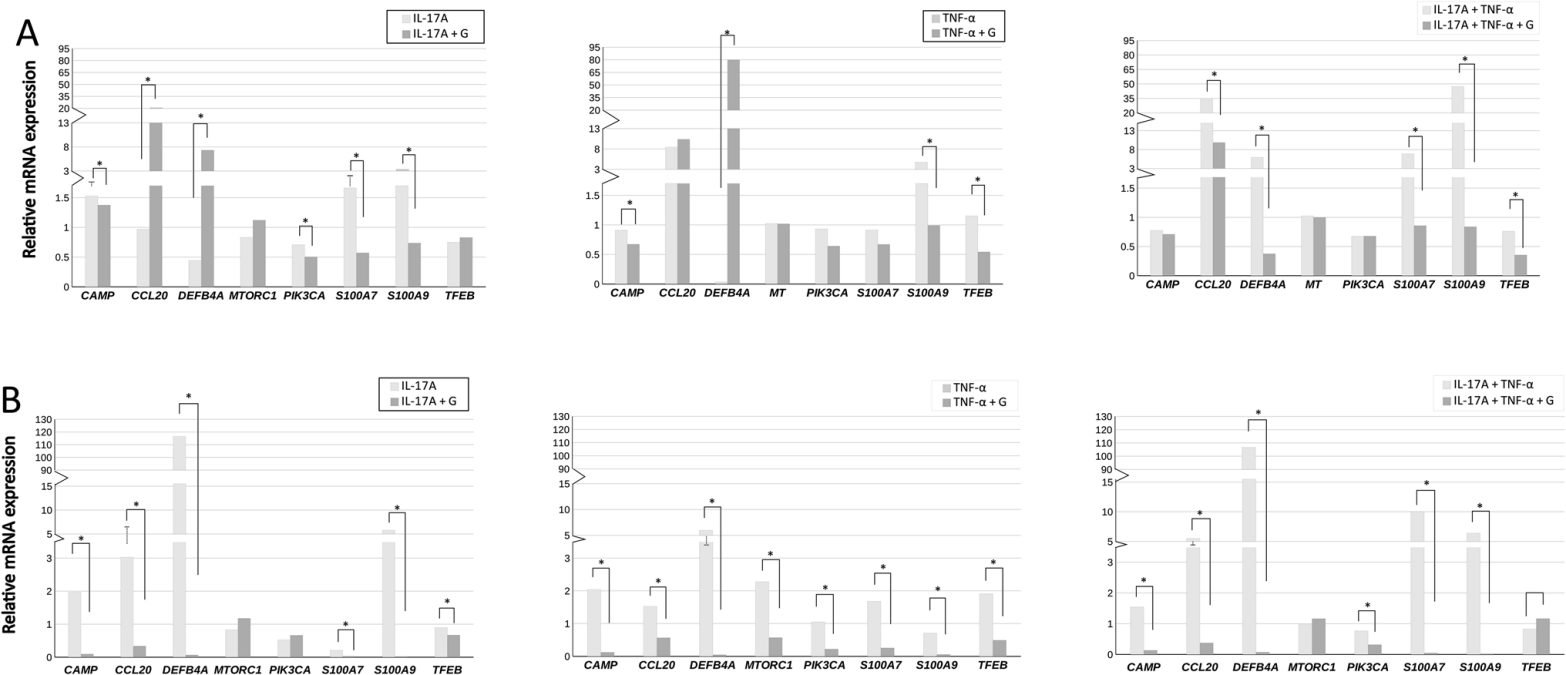
Real-time qRT-PCR verification of selected genes related to the early inflammatory response characteristic of psoriasis (*CAMP*, *CCL20*, *DEFB4A*, *PIK3CA*, *S100A7*, and *S100A9*), as well as *MTORC1* and *TFEB* genes in HaCaT (**A**) and pKC cells (**B**), after the activation with IL-17A, TNF-α, or an IL-17A/TNF-α mix. Keratinocytes were pretreated with 100 μM of genistein for 1 h and then incubated with IL-17A, TNF-α, or an IL-17A/TNF-α mix for the next 24 h. The relative mRNA expression levels were determined against the TATA-Box Binding Protein (*TBP*) reference gene, and the relative quantitation was calculated using ^2−∆∆^Ct. Data represent the mean values ± standard deviation (SD) from 3 independent experiments (*n* = 3). Comparisons among particular paired cells stimulated with IL-17A, TNF-α, or IL-17A/TNF-α mix before and after treatment with genistein were performed, and significant differences were observed following the application of a parametric Student's t-test, with continuity correction (*p* ≤ 0.05). The asterisks designate a statistically significant difference. *IL-17A* interleukin-17A, *TNF-α* tumor necrosis factor-alpha, *G* genistein.

Real-time qRT-PCR analyses of HaCaTs and pKCs revealed that during the development of Ps inflammation induced by IL-17A, TNF-α, or IL-17A/TNF-α mix, the level of mRNA associated with the early inflammatory response characteristic for Ps (*CAMP*, *CCL20*, *DEFB4A*, *PIK3CA*, *S100A7*, and *S100A9*), as well as key regulatory genes (*MTORC1* and *TFEB*), were strongly inflected (Fig. 5A for HaCaTs and B for pKCs). Considering the discrepancy in expression levels between HaCaT and pKCs cells, the results indicated a varied modulation profile of the tested genes. HaCaT stimulation only with IL-17A or TNF-α was not sufficient to induce expression of most of the tested genes associated with the inflammatory response. The most prominent effect was noticed for activation with the IL-17A/TNF-α cytokine mix (Fold Change, FC: 34.6 for *CCL20,* FC: 5.9 for *DEFB4A*, FC: 6.9 for *S100A7*, and FC: 47.4 for *S100A9*). In contrast, the expression of *CCL20* and *DEFB4A* increased even more after genistein treatment in HaCaT stimulated with IL-17A or TNF-α alone. The desired effect of the tested isoflavone was observed for the S100 family genes, for which this compound has an anti-inflammatory effect. For HaCaT cultured with TNF-α or the IL-17A/TNF-α cytokine mix, expression of the *TFEB* gene was also remarkably reduced after genistein pre-incubation. Going forward, our findings suggest that in the pKCs, a permanent increase in *CAMP, CCL20,* and *DEFB4A* gene expression occurred, regardless of the method of stimulation, but the most prominent was observed for the IL-17A/TNF-α mix as it was seen in HaCaTs. The *S100A7* gene was upregulated after TNF-α, as well as IL-17A/TNF-α, mix activation. Consecutively, IL-17A and the IL-17A/TNF-α mix were necessary to activate the expression of the *S100A9* gene. We found that expression of the aforementioned genes was significantly decreased after 1 h pretreatment with genistein, compared with the group treated with IL-17A alone (5 × for *CAMP*; 8 × for *CCL20*; 1628 × for *DEFB4A,* and 213 × for *S100A9*), TNF-α alone (16 × for *CAMP*; 2.7 × for *CCL20*; 118 × for *DEFB4A,* and 6.6 × for *S100A7*), and IL-17A/TNF-α mix (11 × for *CAMP*; 12.9 for *CCL20*; 1473 × for *DEFB4A,* 207 × for *S100A7,* and 273 × for *S100A9*). The expression of *MTORC1* and *PIK3CA* genes was attenuated in pKC cultures stimulated with TNF-α, after 1 h pretreatment with genistein (FC: 0.6 and FC: 0.2, respectively), and *PIK3CA* in the IL-17A/TNF-α mix stimulated cells (FC: 0.3). The expression of the *TFEB* gene decreased in pKCs treated separately with IL-17 + G and TNF-α + G (1.3 × and 3.5x, respectively), but not in cells treated with IL-17A/TNF-α mix + G.

The presented data indicate that the isoflavone applied through the study attenuated the IL-17A, TNF-α, or IL-17A/TNF-α mix-induced pathway at the mRNA level, which was more noticeable for pKCs.

## Discussion

Much research is still devoted to the discovery of new therapeutic anti-psoriatic substances, which are tested in preclinical laboratory studies and clinical phase trials^25^. Among them are natural compounds that may prove to be very important in the treatment of Ps. Their anti-inflammatory and anti-proliferative effects may be complementary to systemic treatment, but there is also a substantive therapy for milder forms of disease. Among the natural compounds, great attention is paid to isoflavones. They are applied worldwide as alternative medicaments for Ps because of their perceived beneficial impact on the skin^26–28^. Lastly, we examined the effects of the isoflavone genistein on activated, spontaneously immortalised human keratinocytes in a HaCaT cell line with with proinflammatory phenotype to find new potential targets for therapy and/or to develop a tool for treatment^29^. Independently, Wang et al.^17^ showed that genistein significantly inhibits the proliferation of HaCaTs with or without tumor necrosis factor-alpha (TNF-α) induction and abolishes 20 ng/ml TNF-α induced inflammatory cytokines and chemokine mRNA upregulated expression, including IL-1β, IL-6, IL-8, IL-23, vascular endothelial growth factor A (VEGF-A), and chemokine (C–C motif) ligand 2 (CCL2). Research done by Hsin Ju-Li et al.^30^ demonstrated that isoflavone extract could also effectively ameliorate IMQ (imiquimod)-triggered Ps-like skin inflammation, reducing skin thickness and redness, as well as the cumulative score in a concentration-dependent manner.

Thus, the objective of this study was to determine the impact of the isoflavone genistein on Ps through in vivo and in vitro tests. The potential use of this compound as monotherapy in patients with mild to moderate plaque Ps covered the evaluation of the efficacy of two dosing regimens of genistein at 75 or 150 mg per day administered orally. Considering the current data, the treatment of Ps patients seems to be a life-long therapy. Thus, the long-term safety of potential drugs is one of the main factors that influences treatment decisions. Accordingly, the primary objective of this study was to determine the safety and tolerability of genistein in cohorts treated with this agent or a placebo. Adverse events (AEs) were minor, and the tested isoflavone was generally well tolerated and safe to use. Over time, there was no increase in the incidence of AEs reported with genistein treatment compared to the placebo. As can be seen in the literature, our results concerning safety and tolerability are consistent with the reports of other researchers testing genistein at similar doses for its application in the treatment of various diseases^31,32^. Next, the pharmacodynamic profile of genistein was assessed by changes in the Psoriasis Area and Severity Index (PASI), Body Surface Area (BSA), Physician’s Global Assessment (PGA), and selected cytokine biomarkers in serum, its activity on functional health and health-related quality of life, and its efficacy compared to the placebo. Cohorts of 34 patients were given all planned doses of the study drug (i.e., 75 or 150 mg per day, or placebo) and completed the study after 56 days. Here, we did find some evidence of genistein-dependent improvement for the Ps clinical severity scores, such as for the PGA score in genistein groups (GEN 75 and GEN 150) and placebo on day 56, which was close to statistical significance (*p* = 0.0506) (Table 1). In addition, the impact of the tested agent on Ps patients in relation to clinical parameters was minutely studied for four of the 34 enrolled subjects, named u.09, 11, 12, and 15, included later in a biochemical analysis (Table 2). In this case, in the absence of statistical calculations, based on our experimental observations, one can infer a reduction of both factors, PASI and BSA, but not PGA, for patients u.09 and 12 among the three genistein-treated subjects. Notably, a regression of the Ps phenotype was visible for patient u.12, based on photo-documented psoriatic lesion evaluation.

In the literature, genistein was demonstrated to influence the expression of inflammatory mediators^33^. However, when investigating the pharmacodynamic effects of this isoflavone on serum biomarkers in our samples, we did not observe statistical significance between and within the studied cohorts (Tables 3 and 4). The pharmacodynamic assessment was evaluated on a panel of four different inflammatory proteins, interleukin (IL)-12, IL-17, IL-23, and TNF-α, at baseline (day 0) and after 56 days of genistein handling. This outcome resulted in the inability to conduct any correlation analysis of inflammatory biomarkers with the clinical parameters of patients participating in this trial. Of course, clinical efficacy was rather poor in our test, which may be related to the fact that high doses of the agent do not result in better effects than low doses due to the limited absorption of isoflavone preparations^16^. In addition, frequent and modest intakes of genistein over the day rather than as a single high dose per day could be more efficient^34^. Although our studies implicate genistein as having a minor impact on the level of inflammatory mediators, one should consider that this study was performed only systemically (serum level) due to the restricted access to a larger quantity of material, so it may be important to examine these factors locally (lesional skin level). Such practise is presented in experiments with polyphenols conducted on rodents based on topical application where disease indicators, such as cytokine levels, are estimated in skin samples but not in serum^35,36^. Yet another aspect of the issue discussed here is the validation of the efficacy of genistein treatment in both oral and transcutaneous ways to achieve an even greater benefit for the skin. It can also have significance for the use of antibacterial properties of genistein in the case of difficult to heal wounds in patients with Ps^37^. In contrast, modulation of the inflammatory response at various tissue levels in a disease such as Ps might be of special profit. Indeed, the tissue-cross consideration regarding the activity assessment of inflammation-immune axis genes in the HaCaT cell line with with proinflammatory phenotype joining the genistein test allowed us to conclude that this isoflavone exhibits potential as an active anti-inflammatory agent, associated with its capacity to modify the pattern of transcript levels across keratinocyte cells (see Fig. 3 in Ref.^29^)^16^. Notably, a number of factors can influence the absorption of bioactive compounds, such as isoflavones. Two parallel studies demonstrated that an intake of 100 mg isoflavones/day resulted in total circulating polyphenols of 1.12 and 4.50 μmol/l^38,39^, but inter-individual variation, estimated based on repeated measures in one of the studies, was 30–96%. In a randomised trial determining whether a soy isoflavone supplement improves asthma control in adolescent and adult patients with poorly controlled disease, the mean plasma genistein level increase was from 4.87 to 37.67 ng/ml (*p* < 0.001) in participants receiving the supplement^40^. Research data from intervention studies in humans, focusing on the factors that affect the bioavailability of soy isoflavones, reported the influence of diet due to interactions between dietary components and the effect on composition of the gut microbiota, which in turn plays a crucial role in isoflavone bioavailability^41–43^. In support of a role for microbial regulation of isoflavone metabolism are studies completed in rats lacking soy metabolising gut microbiota, unable to metabolise isoflavones unless inoculated with bacterial strains^44^. From this point of view, it would be significant to validate genistein serum levels after oral administration in patients enrolled in the present study to compare them with clinical outcomes.

**Figure 6:**
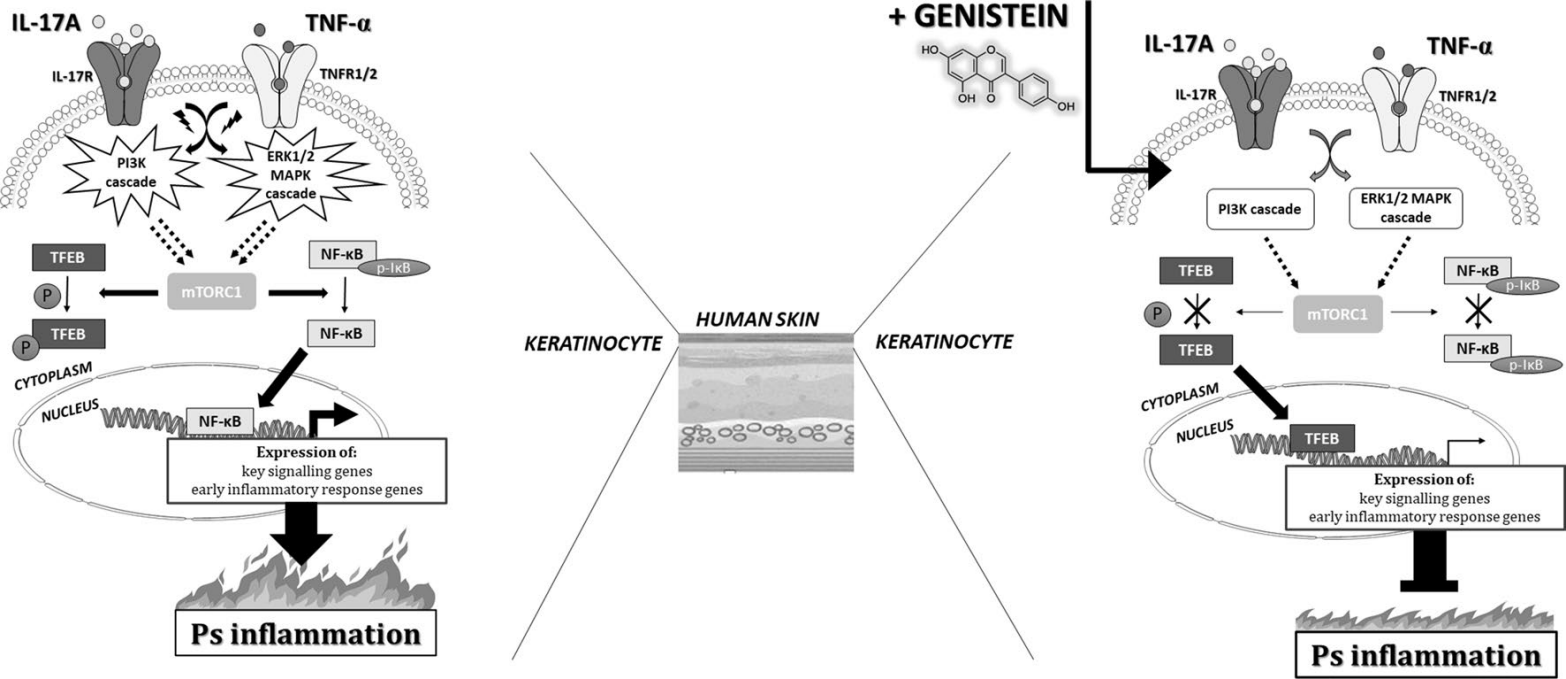
Schematic representation of the molecular basis of genistein action in control of psoriasis inflammation. *IL-17A* interleukin-17A, *IL-17R* interleukin-17 receptor, *TNF-α* tumor necrosis factor-alpha, *TNFR1/2* TNF receptor-1/2, *PI3K cascade* phosphoinositide 3-kinase cascade, *ERK1/2 MAPK cascade* mitogen-activated protein kinase relevant extracellular signal-regulated kinase 1/2 cascade, *TFEB* transcription factor EB, *P* phosphorus, *mTORC1* mammalian targets of rapamycin complex 1, *NF-κB* nuclear factor kappa-light-chain-enhancer of activated B cells, *p-IκB* phosphorylase inhibitor of kappa B, *Ps inflammation* psoriasis inflammation. Figure created using the receptors, human skin and flame graphs designed by brgfx/Freepik (www.freepik.com) and Servier Medical ART/SMART (https://smart.servier.com).

Our follow-up in vitro studies demonstrated that, in human keratinocytes, both human adult low calcium high temperature cells (HaCaTs) and primary keratinocytes (pKCs), cytokines, such as IL-17A, TNF-α, and their mix, modulate to some extent mitogen-activated protein kinase (MAPK) pathway phosphorylation, nuclear factor kappa-light-chain-enhancer of activated B cells, p65 subunit (NF-κB p65 subunit) position, and Ps-inflammatory response gene expression status (Figs. 3, 4, 5). The results of the analyzes showed more or less subtle discrepancies with the use of both cell lines due to specific differences and limitations of tested keratinocytes, which we discussed in detail in our previous reports^25,45^. Thus, the studies of Ps based on HaCaT and pKC cells as experimental models shall take this aspect into account. Indeed, several signalling pathways have been noted to be abnormally activated in Ps. In normal conditions, activated MAPKs interact with and phosphorylate numerous cytoplasmic substrates and ultimately modulate transcription factors that drive context-specific gene expression. Defects in these processes, in turn, have long been hypothesised to participate in the pathology of Ps, yet their implication in the altered psoriatic molecular profile remain elusive. In the MAPK pathway, the keratinocytes exposed to IL-17A and genistein combination revealed a substantial decrease in extracellular signal-regulated kinase 1/2 (ERK1/2) phosphorylation status, which was upregulated by IL-17A (Fig. 3A). Furthermore, IL-17A was also responsible for the increase of phosphatidylinositol 3-kinase (PI3K) phosphorylation in pKCs, significantly reducing it in response to genistein (Fig. 3B). At the same time, we observed considerable downregulation of the *PIK3CA* gene, which provides instructions for making the p110α protein (an action subunit of PI3K) in response to genistein in HaCaTs stimulated with IL-17A, similar to the case of TNF-α and mixture of IL-17A/TNF-α-induced pKCs (Fig. 5). This is consistent with our previous study (see S5 Table in^29^), which was also confirmed in our subsequent research^16^. The inflammatory and proliferative cascades of psoriatic disease also concern PI3K downstream pathways, including mammalian targets of rapamycin (mTOR) signalling, as well as mTOR complex 1 (mTORC1)-dependent nuclear import of transcription factor EB (TFEB). TFEB adjacent to master growth regulator mTORC1 remains in a phosphorylated inactive form in the cytoplasm in the cell, whereas inhibition of mTORC1 by genistein, as reported previously in the literature, may activate TFEB by promoting its nuclear translocation, resulting in stimulation of certain genes’ expression. In addition, in our previous research, unrelated to Ps, we found a regulatory network linking genistein-mediated control of *TFEB* expression and TFEB nuclear translocation^47^. Thus, both *MTORC1* and *TFEB* activities were examined in the course of the current investigations, and thereby we found that genistein remarkably suppressed the mRNA level of *MTORC1* in pKCs stimulated by TNF-α and *TFEB* in TNF-α and IL-17A/TNF-α mix-induced HaCaTs and IL-17A, TNF-α, and IL-17A/TNF-α mix-induced pKCs (Fig. 5). In addition, growing evidence has shown that NF-κB is involved in other cellular signalling pathways becoming activated and is overexpressed in skin tissues in Ps^47^. Indeed, this was consistent with our current study and proved by statistically significant TNF-α and IL-17A/TNF-α mix or IL-17A, TNF-α, and IL-17A/TNF-α mix dependent NF-κB p65 subunit cytoplasm-to-nucleus shuttling in HaCaT and pKC cells, respectively (Fig. 4). In addition, this effect was remarkably reversed by genistein. Consistently, the isoflavone could substantially inhibit TNF-α and IL-17A/TNF-α mix induced nuclear translocation of the NF-κB p65 subunit in HaCaTs after 1 h, as well as after 1 and 24 h for TNF-α and 1 h IL-17A/TNF-α mix in pKCs (Fig. 4). In addition, our previous studies alluded the effect of genistein on NF-κB nuclear transfer (see Fig. S4 in^29^). In NF-κB, as well as the ERK1/2 MAPKs signalling modules, which mediate extracellular signals in the nucleus to turn on the responsive genes in cells, being regulated by cytokines could be responsible for modulation of the activity of genes that are critical for inflammatory responses. This may also apply to other important regulatory genes in the cell, such as *MTORC1* and *TFEB*. Considering that, it was reasonable to examine mRNA levels of key genes selected from this group. Our real-time qRT-PCR analyses of HaCaT and pKC cells displayed that during the development of psoriatic inflammation status (induced by IL-17A, TNF-α, or their mix), regulated levels of the gene transcripts triggering the host early inflammatory response characteristic for Ps (i.e., *CAMP*, *CCL20*, *DEFB4A*, *S100A7*, and *S100A9)* were detected (Fig. 5). Moreover, we found that up- or downregulation of expression of most of these genes decreased significantly after keratinocyte pretreatment with genistein assisted by stimulation with IL-17A, TNF-α, or their mix in both cell lines tested. The main function of cathelicidin CAMP, chemokine CCL20, defensin DEFB4A, and S100 proteins, concentrated on the human skin surface, is to destroy pathogenic microorganisms. In agreement with a role for these peptides in antimicrobial immunity, Ps lesions are rarely infected. In patients with Ps lesions, these molecules are substantially expressed and are considered to play an important role in the pathogenesis of Ps^48^. Our results indicate that genistein attenuated the IL-17A, TNF-α, or IL-17A/TNF-α mix-induced inflammation pathway at the mRNA level of the tested genes. The cellular pathways are highly complex; thus, a large degree of crosstalk within the MAPK cascades, PI3K, NF-κB pathways, and other signalling networks exists. Schematic representation of the molecular basis of genistein action in control of psoriasis inflammation is shown in Fig. 6. All our data demonstrate that genistein can effectively suppress the activation of various cellular signalling cascades with variable but limited effects. Therefore, it could be reasonable to postulate the medical use of genistein as a combination therapy.

In summary, we concluded that the benefits brought by the tested isoflavone in the immense panorama of Ps therapies are above others in terms of safety. Therapeutically, genistein represents a promising option, as it is well tolerated and no blood test or imaging exam is mandatory during treatment. Another important advantage of this agent is the route of administration and the possibility of it being self-administered. Genistein may offer an oral treatment option for those patients who discontinue treatments because of ineffectiveness, intolerability, or ineligibility to the currently available drugs. As is increasingly observed, another aspect of the medical use of genistein is its contribution to a combination therapy. Combination therapies, as compared to single drug therapy, are considered effective if they provide a better response together than the individual drugs by themselves. Many drug combinations can be additive or even synergistic, so further research into the effects of the medication(s) and genistein combination remains essential. Thus, to place genistein in the Ps therapeutic algorithm, more multifaceted, as suggested above, head-to-head trials testing this agent versus other therapeutics may become valuable. In addition, the hypothesis presented in this report will be verified in the future with an appropriate 2D co-culture, 3D organotypic tissue, and also with an animal model displaying Ps phenotype.

## Materials and methods

### In vivo experiments

#### Ethical approval and study design in reference for blood sampling

The studies were conducted on blood samples originated from the clinical trial reported in^16^, in compliance with the Declaration of Helsinki, the Good Clinical Practice Guidelines established by the International Conference on Harmonisation, and local country regulations relevant to the use of new therapeutic agents. In the final protocol, all amendments and informed consent documentation related to testing of samples derived from the clinical trial were reviewed and approved by Independent Ethics Committee at the Regional Chamber of Physicians in Warsaw, under the number KB/910/13. Patients provided written informed consent. They were ≥ 18 years old with a diagnosis of mild to moderate chronic plaque psoriasis (Ps) for ≥ 12 months prior to the first dose of the study drug. At baseline, patients had to have a Psoriasis Area and Severity Index (PASI) score ≤ 12, a Physician’s Global Assessment (PGA) score of 2 or 3, and < 10% Body Surface Area (BSA) Ps involvement. Eligible patients complied with prohibited medications and phototherapy requirements as follows: (i) cyclosporin A, methotrexate, retinoids, biological drugs, and oral and topical glucocorticosteroids were prohibited 6 weeks before the first administration of the study product until study completion; and (ii) exposure to UVA and UVB light was prohibited 6 weeks prior to the first administration of the study product until the study completion. Other exclusion criteria were concomitant renal, gastrointestinal or haematological disease, a history of any malignancy, pregnancy, or breast feeding, and clinically significant abnormal laboratory values in haematology, blood chemistry, or urine analysis. The results of the original trial were identified and described in our reference^16^.

#### Safety assessments

**Figure 7:**
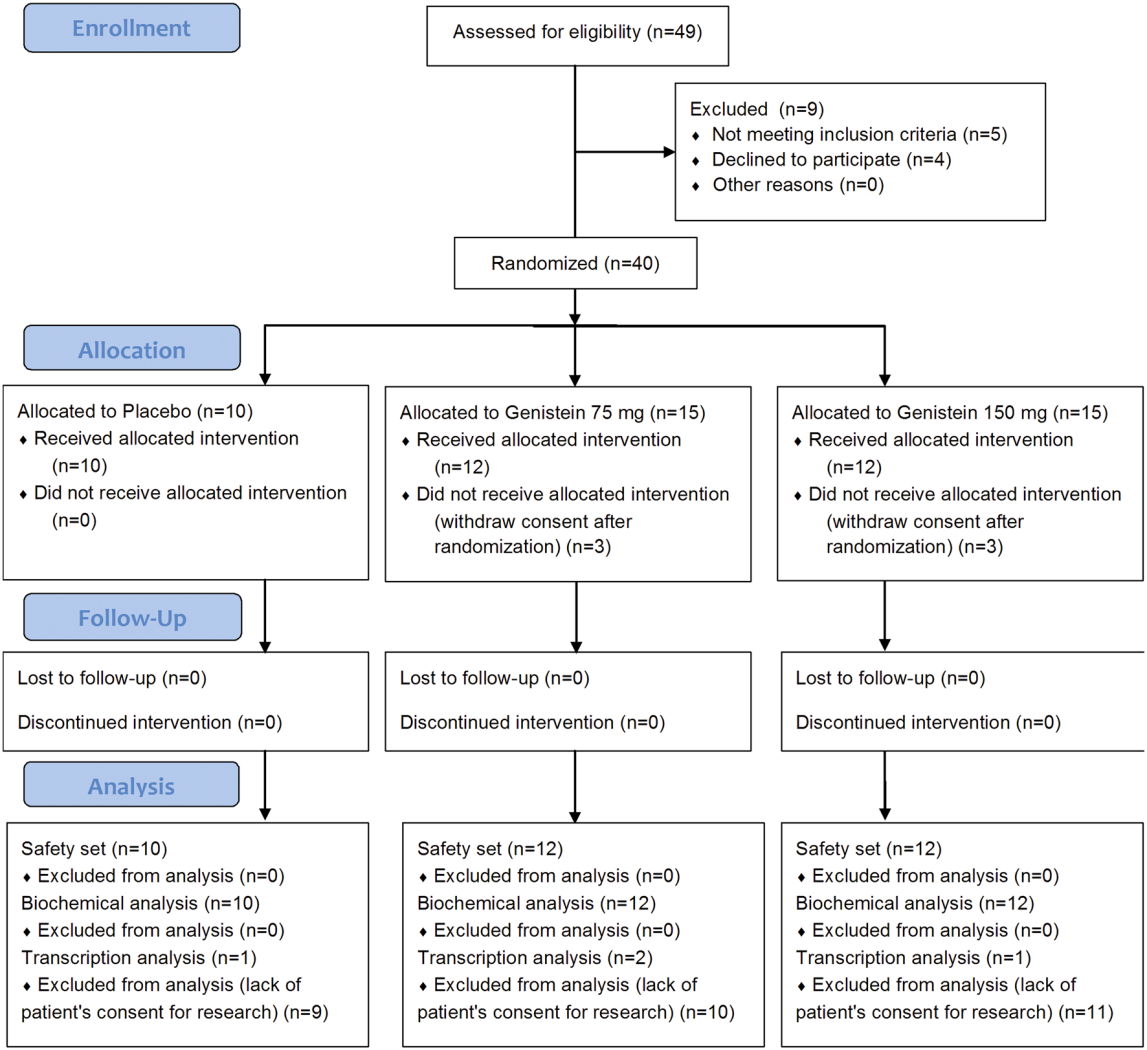
Overview of in vivo study related to the clinical trial reported in^16^.

Safety was evaluated based on vital signs (weight, blood pressure, pulse, temperature, and respiratory rate) and physical examination on all dose administration and evaluation days. A neurological exam and laboratory tests (urinalysis, serum chemistry and haematology) were performed on days 0 and 56. Genistein dosing and blood preparation for pharmacodynamics profile evaluation: patients were screened 1–21 days prior to the administration of the first dose of genistein or placebo on day 0. The study was a multicentre, randomised, double-blind, placebo-controlled, two-dose investigation. In a prospective stage, 34 patients with active mild to moderate chronic plaque Ps were treated under fasting conditions with oral film-coated tablets containing two doses of 75 or 150 mg genistein (GEN), synthesised at the Pharmaceutical Research Institute (Warsaw, Poland), per day (Fig. 7). Blood samples were collected at two time points: baseline (day 0) and after 56 days (day 56) of therapy. For pharmacodynamics and sandwich enzyme-linked immunosorbent assays (ELISA) assessments, blood samples were collected into 8 ml CPT Vacutainer tubes (BD Biosciences) and centrifuged at 1500 × *g* for 20 min at room temperature within 2 h of blood collection. Serum was harvested as 4 aliquots, frozen on dry ice, and stored at -80 °C.

#### ELISA

The levels of interleukin (IL)-12 p70, IL-17F, IL-23, and tumor necrosis factor-alpha (TNF-α) were determined in the serum of the patients. Samples were stored frozen in small aliquots (500 µl) and thawed once. The quantification of cytokines was performed using the following commercially available sandwich enzyme-linked immunosorbent assays (ELISA), according to the manufacturer’s instructions: human IL-12 p70, human IL-17F, human IL-23, and human TNF-α ELISA kit Promokine (PromoCell GmbH, Heidelberg, Germany). Each serum sample was assayed twice, and cytokine levels were determined with the spectrophotometer using the 450 nm wavelength. According to the information provided by the manufacturer of the ELISA kits, the lower detection limits for IL-12 p70, IL-17F, IL-23, and TNF-α were 2.1, 20, 10, and 30 pg/ml, respectively.

### In vitro experiments

#### Primary human keratinocyte isolation, cell line, and culture

Human adult low calcium high temperature cells (HaCaT), spontaneously transformed keratinocytes from histologically normal skin, were purchased from the CLS Cell Lines Service GmbH (Eppelheim, Germany). HaCaT cells from passage 40–43 were maintained in DMEM (Gibco, Thermo Fisher Scientific, Waltham, MA, USA) supplemented with 10% FBS (Gibco, Thermo Fisher Scientific, Waltham, MA, USA) and 1% A/A solution (Gibco, Thermo Fisher Scientific, Waltham, MA, USA) at 37 °C.

Primary human epidermal keratinocytes (pKCs) were assembled within the framework of our collaboration with the Department of Dermatology, Venerology, and Allergology at the Medical University of Gdańsk, and signed informed consent was obtained from all subjects under protocols approved by the Independent BioEthics Committee of the Medical University of Gdańsk (NKBBN/161-634/2018). They were isolated from normal epidermises separated by 10 U/ml Dispase II (Sigma-Aldrich, St. Louis, MO, USA) from the dermis and treated with 0.05% trypsin/EDTA (Gibco, Thermo Fisher Scientific, Waltham, MA, USA) to obtain a single cell suspension. The number of viable cells was determined using a Scepter 2.0 Cell Counter (Merck Millipore, Burlington, MA, USA). Primary keratinocytes were seeded at a density of 5 × 10^6^ cells/cm^2^ in Dulbecco’s Modified Eagle’s Medium (DMEM, Gibco, Thermo Fisher Scientific, Waltham, MA, USA) supplemented with 10% fetal bovine serum (FBS, Gibco, Thermo Fisher Scientific, Waltham, MA, USA) and 1% antibiotic–antimycotic (A/A, Gibco, Thermo Fisher Scientific, Waltham, MA, USA) at 37 °C. After 24 h, the medium was changed to EpiGRO™ medium supplemented with L-Glutamine, EpiFactor P, Epinephrine, rh TGF-α, Hydrocortisone hemisuccinate, rh Insulin, Apo-Transferrin (Merck Millipore, Burlington, MA, USA) and 1% A/A solution (Gibco, Thermo Fisher Scientific, Waltham, MA, USA).

For stimulation of HaCaT and pKC, cell cultures were carried out until approximately 70% confluence was achieved. After this time, keratinocytes were enzymatically digested using 0.05–0.25% trypsin–EDTA (Gibco, Thermo Fisher Scientific, Waltham, MA, USA) and seeded on 6-well plates at a density of 5 × 10^4^ cells/cm^2^ in serum-free medium (SFM) with the addition of 25 mg bovine pituitary extract (BPE), 2.5 µg epidermal growth factor (EGF) (Gibco, Thermo Fisher Scientific, Waltham, MA, USA), and 1% A/A solution (Gibco, Thermo Fisher Scientific, Waltham, MA, USA). Following a 24 h incubation, the medium was changed to SFM without growth supplements for a further 16 h. One hour before activation, the cells were preincubated with 100 μM genistein (Pharmaceutical Research Institute, Warsaw, Poland) or 0.05% dimethyl sulfoxide (DMSO, Sigma-Aldrich, St. Louis, MO, USA) as a control. Next, cells were stimulated with IL-17A (Abcam, Cambridge, United Kingdom) or TNF-α (Abcam, Cambridge, United Kingdom) alone or with IL-17A/TNF-α mix (Abcam, Cambridge, United Kingdom) at a concentration of 100 ng/ml, 10 ng/ml, and 100/10 ng/ml, respectively. Keratinocytes were cultured for 24 h and then used for further analysis.

#### PI3K phosphorylation analysis by the Guava® Muse® cell analyser

The MUSE® PI3K Activation Dual Detection Kit (Luminex, Austin, TX, USA) was used to detect Akt phosphorylation (Ser473) relative to the total Akt expression in one sample. HaCaT and pKC cells were seeded at a density of 5 × 10^4^ cells/cm^2^ into 6-well plates in SFM supplemented with bovine pituitary extract (BPE) and epidermal growth factor (EGF) (Gibco, Thermo Fisher Scientific, CA, USA). After 24 h, the culture medium was replaced with SFM without growth supplements for a 16 h incubation. For further experiments, cells were pretreated with 100 μM genistein for 1 h and then incubated with IL-17A and TNF-α alone or with a combination of both (Abcam, Cambridge, United Kingdom) for 3 h. The same conditions were conducted with the addition of 100 μM genistein (Pharmaceutical Research Institute, Warsaw, Poland). DMSO (0.05%) or wortmannin (50 nM) was added to the control cells, or they were left untreated. Trypsinised and suspended cells were collected, and the phosphorylation of PI3K was determined by the Guava® Muse® Cell Analyser (Luminex, Austin, TX, USA) using commercial kit Muse® PI3K Activation Dual Detection Kit (Luminex, Austin, TX, USA) according to the manufacturer’s instructions. An average of 10,000 cells were analysed for each condition. Duplicate independent experiments were conducted. The percentage of inactivated cells, activated cells (via PI3K phosphorylation), and non-expressing cells was determined for each experimental condition.

#### MAPK activation assay by the Guava® Muse® cell analyzer

MAPK phosphorylation was evaluated relative to the total MAPK expression by the MUSE® MAPK Activation Dual Detection Kit (Luminex, Austin, TX, USA), including phospho-specific anti-phospho-ERK1/2 (Thr202/Tyr204, Thr185/Tyr187)-phycoerythrin and anti-ERK1/2-PECy5-conjugated antibodies. Therefore, phosphorylated and non-phosphorylated proteins were detected simultaneously in each sample, as well as their ratio, allowing a more accurate normalisation process. HaCaT and pKC cells were seeded at a density of 5 × 10^4^ cells//cm^2^ into 6-well plates in SFM supplemented with BPE and EGF (Gibco, Thermo Fisher Scientific, CA, USA). After 24 h, the culture medium was replaced with SFM without growth supplements for a 16 h incubation. For further experiments, cells were pretreated with 100 μM genistein (Pharmaceutical Research Institute, Warsaw, Poland for 1 h and then incubated with IL-17 and TNF-α alone or a combination of both (Abcam, Cambridge, United Kingdom) for 3 h. The control cells were treated with 0.05% DMSO or were left untreated. Trypsinised and suspended cells were collected, and the phosphorylation of MAPK was determined by the Guava® Muse® Cell Analyser (Luminex, Austin, TX, USA) using commercial kit Muse® MAPK Activation Dual Detection Kit (Luminex, Austin, TX, USA) according to the manufacturer’s instructions. An average of 10,000 cells were analysed for each condition. Duplicate independent experiments were conducted.

#### NF-κB translocation assessment by immunofluorescence

To detect the nuclear trafficking of the nuclear factor kappa-light-chain-enhancer of activated B cells, p65 subunit (NF-κB p65 subunit) 2 × 10^4^ HaCaT and pKC cells were seeded in chamber slides (Millicell EZ SLIDES, Merck Millipore, Billerica, MA, USA) in supplemented SFM (Gibco, Thermo Fisher Scientific, CA, USA). After a 24 h period, cells were maintained in SFM without growth supplements for 16 h. For the experimental procedure, the cells were pretreated with 100 μM genistein (Pharmaceutical Research Institute, Warsaw, Poland) for 1 h and then incubated with IL-17A (100 ng/ml), TNF-α (10 ng/ml), or a mix of both (Abcam, Cambridge, United Kingdom) for the next 24 h. Concurrently, all conditions were examined with the addition of 100 μM genistein. The control cells were treated with 0.05% DMSO.

For immunostaining, cells were fixed with 4% paraformaldehyde for 15 min, permeabilised in 0.1% Triton X-100 for 30 min, blocked with 3% BSA/0.1% Triton X-100 for 1 h, and further incubated overnight at 4 °C with rabbit monoclonal antibody NF-κB p65 (D14E12) XP® Rabbit mAb (1:400, Cell Signaling Technology, Danvers, USA) in blocking buffer. Fluorescently tagged secondary antibodies, anti-rabbit IgG (H + L), F(ab’)2 Fragment (1:250, Alexa Fluor® 488 Conjugate, Cell Signaling Technology, Danvers, USA) were then administered for 2 h at room temperature in the dark. Nuclei were counterstained with 4′,6-diamidino-2-phenylindole (DAPI) (Slow Fade Diamond Antifade Mountant with DAPI, Invitrogen, California, USA) for 1 h in the dark. Visualisation was performed with a fluorescent microscope (Leica DMI4000B) at a 100 × magnification. Results representative of three independent experiments (with scale bars 100 μm) are shown.

#### RNA processing

Total RNA was extracted from cells using the High Pure RNA Isolation Kit (Roche Applied Science, Penzberg, Germany) following the manufacturer’s instructions. The quantity of each RNA sample was evaluated using a Quant-iT™ RiboGreen™ RNA Assay Kit (Invitrogen, California, USA). The quality of each RNA sample was assessed by agarose gel electrophoresis. The synthesis of cDNA from an RNA template was conducted using a Transcriptor First-Strand cDNA Synthesis Kit (Roche Applied Science, Penzberg, Germany).

#### Gene expression analysis by real-time quantitative reverse transcriptase-polymerase chain reaction (real-time qRT-PCR)

Analysis was carried out with Single Tube Custom TaqMan Gene Expression Assays (Thermo Fisher Scientific, Waltham, MA, USA) and the LightCycler 480 Probes Master (Roche Applied Science, Penzberg, Germany) using the Light Cycler 480 II detection system (Roche Applied Science, Penzberg, Germany). Expression values were normalised against control gene TATA-Box Binding Protein (*TBP)*. The TaqMan probes used were as follows: CAMP (Hs00189038_m1), CCL20 (Hs00355476_m1), DEFB4A/B (Hs00823638_m1), MTORC1 (Hs00234508_m1), PIK3CA (Hs00907957_m1), S100A7 (Hs01923188_u1), S100A9 (Hs00610058_m1), TBP (Hs00427620_m1), and TFEB (Hs00292981_m1). Each experiment of real-time qRT-PCR analysis was repeated triple (*n* = 3). Data are reported as the mean ± standard deviation (SD) with *p* < 0.05 considered statistically significant.

### Statistical analyses for in vivo and in vitro studies

Unless otherwise indicated, data are expressed as the mean ± standard deviation (SD) of at least three independent experiments, using the Statistica Program 13.3 software (Statistica Software, StatSoft Polska).
